# Shift of Graft-Versus-Host-Disease Target Organ Tropism by Dietary Vitamin A

**DOI:** 10.1371/journal.pone.0038252

**Published:** 2012-05-30

**Authors:** Christian Koenecke, Immo Prinz, Anja Bubke, Alina Schreder, Chun-Wei Lee, Oliver Pabst, Reinhold Förster

**Affiliations:** Institute of Immunology, Hannover Medical School, Hannover, Germany; Institut Jacques Monod, France

## Abstract

Gut-homing of donor T cells is causative for the development of intestinal GvHD in recipients of allogeneic hematopoietic stem cell transplantation (HSCT). Expression of the gut-specific homing receptors integrin-α4β7 and chemokine receptor CCR9 on T cells is imprinted in gut-associated lymphoid tissues (GALT) under the influence of the vitamin A metabolite retinoic acid. Here we addressed the role of vitamin A deficiency in HSCT-recipients for donor T cell migration in the course of experimental GvHD. Vitamin A-deficient (VAD) mice were prepared by feeding them a vitamin A-depleted diet. Experiments were performed in a C57BL/6 into BALB/c model of acute GvHD. We found that expression of integrin-α4β7 and CCR9 in GALT was reduced in VAD recipients after HSCT. Competitive *in vivo* homing assays showed that allogeneic T cells primed in VAD mice did not home as efficiently to the intestine as T cells primed in mice fed with standard diet (STD). The course of GvHD was ameliorated in VAD HSCT-recipients and, consequently, their survival was prolonged compared to recipients receiving STD. However, VAD-recipients were not protected and died of clinical GvHD. We found reduced numbers of donor T cells in the intestine but increased cell counts and tissue damage in other organs of VAD-recipients. Furthermore, we observed high IFN-γ^+^CD4^+^ and low FoxP3^+^CD4^+^ frequencies of total donor CD4^+^ T cells in VAD as compared to STD recipients. Taken together, these results indicate that dietary vitamin A deficiency in HSCT-recipients changed target organ tropism in GvHD but also resulted in fatal inflammation after HSCT.

## Introduction

Graft-versus-host-disease (GvHD) is a frequent complication after allogeneic hematopoietic stem cell transplantation (HSCT). Acute GvHD results from an aggressive immune response of alloreactive donor T cells directed against host tissue and affects mostly liver, lung, skin and intestine [1]. Intestinal GvHD can involve any location throughout the gastrointestinal tract and is associated with high morbidity and mortality. Therefore, inhibition or reduction of intestinal GvHD is likely to significantly improve patient’s health and survival. Key events in the development of intestinal GvHD are the generation of alloreactive T cells with gut homing potential and the recruitment of allogeneic effector T cells to the intestinal tract [1]. Shortly after allogeneic HSCT, naïve donor T cells enter secondary lymphoid organs (SLO). After heavy alloantigen-induced proliferation, primed and activated donor T cells leave SLO and enter the host’s organs where they induce severe tissue injury [2]. Thus, inhibition of either the generation of gut-homing T cells or preventing their access to the intestine should counteract the development of intestinal GvHD [3].

Under homeostatic, i.e. non-inflammatory conditions T cell homing into the intestine is regulated by selective interactions of intestinal homing molecules expressed on the surface of T cells and their corresponding ligands expressed in the intestinal mucosa. The integrin-α4β7 is the main adhesion molecule required for lymphocyte entry into the gut-associated lymphoid tissues (GALT), such as mesenteric lymph nodes (mLN) and Peyer’s Patches (PP) and also into the intestinal lamina propria [4]. Furthermore, expression of CC chemokine receptor 9 (CCR9) on T cells directs these cells to the small intestine [5]. Integrin-α4β7 interacts specifically with its ligand mucosal addressin cell adhesion molecule-1 (MAdCAM-1) on intestinal microvascular endothelium, whereas the CC chemokine ligand 25 (CCL25), which is a ligand for CCR9, is selectively expressed in the mucosa of the small intestine but not the colon [6]. Expression of α4β7-integrin and CCR9 are selectively induced during naïve T cell activation in mLN and GALT [7]. In this process, the vitamin A metabolite retinoic acid (RA) has been identified as the central mediator regulating the expression of integrin-α4β7 and CCR9 on T cells in mLN and GALT [8]. The predominant sources of RA seem to be local dendritic cells (DCs) [8], epithelial [9] and stroma cells [10]. In contrast to physiological steady state conditions, T cell homing to the inflamed intestine is not entirely understood. The relevance of integrin-α4β7 expression on donor T cells for intestinal GvHD has been demonstrated [11], [12], whereas the role of CCR9 expression during acute GvHD is unclear.

Based on all these observations we hypothesized that gut-homing of donor T cells during GvHD is likely to be dependent on dietary vitamin A since its metabolite RA potentially induces expression of integrin-α4β7 and CCR9 on allogeneic T cells. Accordingly, a lack of RA should reduce the ability of donor T cells to migrate to the intestine and thus protect from intestinal GvHD. In this study, we thus addressed the role of vitamin A deficiency in HSCT recipients in the course of experimental GvHD. We examined the contribution of dietary vitamin A to the induction of gut-homing molecules on allogeneic T cells in lymphoid organs and their subsequent role during T cell entry to target tissues of acute GVHD. We found that the induction of gut-homing potential of allogeneic donor T cells relied on vitamin A and that vitamin A deficiency in recipient mice prolonged survival from acute GvHD. However, dietary lack of vitamin A did not prevent GvHD and all mice ultimately died of clinically evident disease. We observed increased frequencies of IFN-γ-producing CD4^+^ T cells and reduced FoxP3^+^ regulatory T cells (Treg) in GvHD target organs of VAD recipients. This finding was particularly associated with strong T cell influx and tissue damage of the liver under vitamin A-deficient conditions.

## Results

### Donor T Cell Expression of CCR9 and Integrin-α4β7 During GvHD Priming Phase is Dependent on Dietary Vitamin A

**Figure 1:**
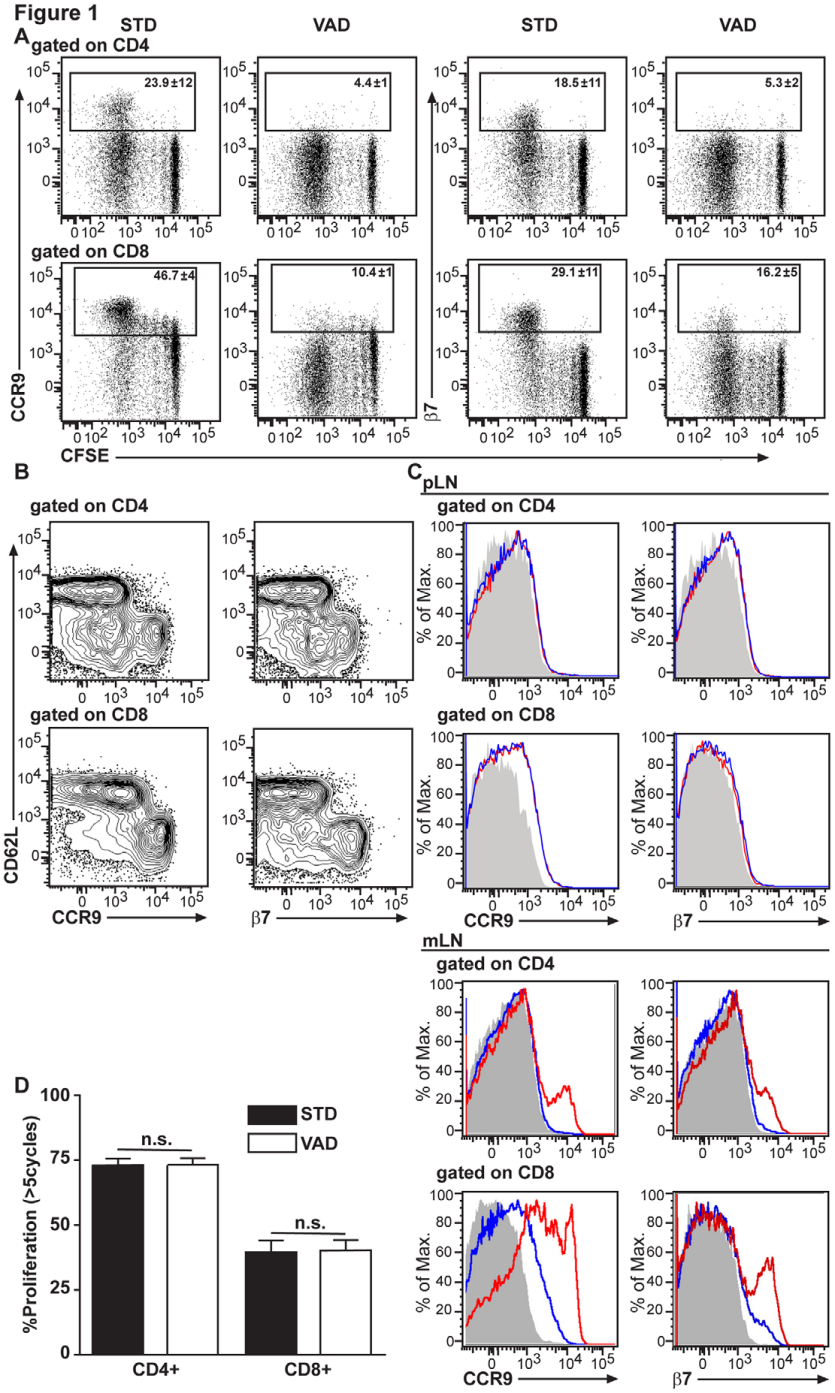
Donor T cell primed in mesenteric lymph nodes express high levels of gut-homing molecules in recipients under standard (STD) but not under vitamin A deficient (VAD) food. (**A**) The expression of CCR9 and integrin-β7 of allogeneic C57BL/6 Thy1.1^+^ CFSE-labeled CD4^+^ and CD8^+^ T cells was determined by flow cytometry three days following adoptive transfer of 2×10^7^ splenocytes into lethally irradiated recipients (BALB/c) fed with STD or VAD diet (N = 3/group). The numbers in the boxes indicate the mean percentage of gated cells +/−SD. Similar results were obtained in at least three repeat experiments. (**B**) Analysis of donor T cells three days after transfer showed an activated phenotype (CD62L^−^) of CCR9^+^ or β7^+^ T cells. Representative data from one of two experiments are shown. (**C**) Expression of CCR9 and integrin-β7 of allogeneic C57BL/6 Thy1.1^+^ CD4^+^ and CD8^+^ T cells in mLN and pLN (grey = isotype control, red = STD recipient, blue = VAD recipient). Similar results were obtained in at least three repeat experiments. (**D**) The proliferation of CFSE-labelled CD4^+^ and CD8^+^ T cells was determined by flow cytometry three days following adoptive transfer of 2×10^7^ splenocytes into lethally irradiated STD or VAD recipients. N = 6/group. Combined data from two experiments (n.s. = not significant).

Allogeneic T cells enter intestinal target sites following their activation and proliferation in GALT [2]. In order to identify the homing molecules that enabled donor T cells to migrate to the inflamed intestine during acute GvHD, we adoptively transferred allogeneic donor T cells into irradiated recipient mice and examined expression of adhesion molecules during allogeneic T cell priming *in vivo*. Thy1.1^+^ C57BL/6 donor T cells were labeled with CFSE and transferred into irradiated recipient BALB/c mice. Three days later, allogeneic donor T cells had undergone more than five divisions in the recipients’ SLOs, which is in line with published data [13]. In mLN, CCR9 and α4β7-integrin were induced at high levels on both donor CD4^+^ and CD8^+^ T cells (**Figure 1A, C**). The pattern of rapid acquisition of CCR9 and β7 on CD4^+^ and CD8^+^ donor T cells after several rounds of cell proliferation was similar to previous observations in experimental GvHD [13]. As expected, the majority of CCR9^+^ and α4β7-integrin^+^ donor CD4^+^ and CD8^+^ T cells were CD62L negative (**Figure 1B**).

Next, we raised BALB/c mice from gestational day 14 onward with a diet that lacked vitamin A and then used these VAD animals as recipients for bone marrow transplantation to determine the influence of dietary vitamin A on expression of gut-homing molecules such as CCR9 and integrin-α4β7 on allogeneic T cells after bone marrow transplantation.

Three days after transplantation, we observed significantly lower induction of CCR9 and integrin-α4β7 on CD4^+^ donor T cells in GALT of VAD recipient mice when compared to mice fed with STD (**Figure 1A**). The same effect was observed on CD8^+^ donor T cells for CCR9 and integrin-α4β7 (**Figure 1A**). This observation was not seen in peripheral lymph nodes (pLN) where expression of these molecules was low and independent of the type of diet fed (**Figure 1C**). Of note, the proliferation of donor T cells in mLN was not affected in VAD recipient mice as determined by CFSE dilution (**Figure 1D**).

### Homing of Allo-primed T Cells to the Intestine is Dependent on Dietary Vitamin A

**Figure 2:**
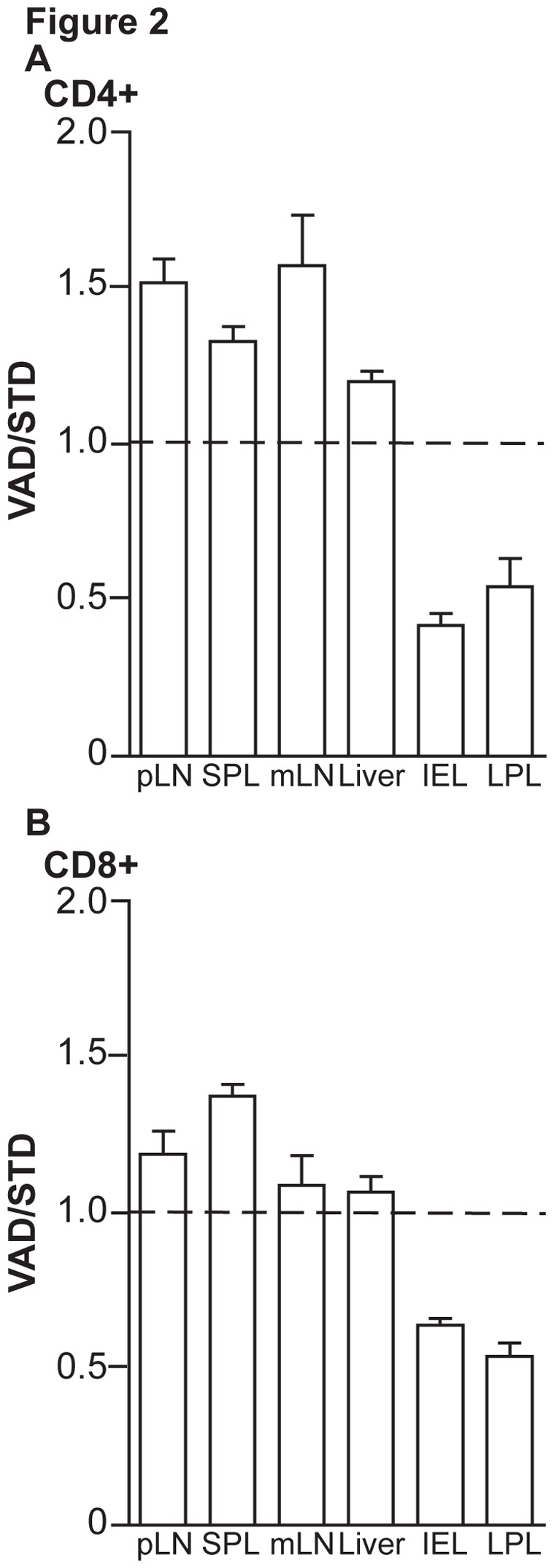
Homing of allo-primed T cells to the intestine is dependent on dietary vitamin A. 2×10^7^ splenocytes (C57BL/6) were transferred into lethally irradiated STD or VAD BALB/c recipients. Three days after transfer donor T cells from mesenteric lymph nodes of STD or VAD BALB/c mice were harvested and then split and differentially labeled with either TAMRA or CFSE. CFSE-labeled cells from STD recipients were mixed with TAMRA-labeled cells from VAD-recipients at a 1∶1 ratio. In cross-labeling experiments, TAMRA-labeled cells from STD-recipients and CFSE-labeled cells from VAD-recipients were used. Mixtures of 5×10^6^ T cells per mouse in total were injected into the tail vein of untreated wt C57BL/6 recipient mice. Eighteen hours after transfer recipient mice were sacrificed and the homing of CD4^+^ and CD8^+^ T cells was analyzed by flow cytometry. The ratio of transferred T cells primed in allogeneic VAD versus STD recipients was analyzed in pLN (pooled per mouse), SPL, mLN, liver, IEL and LPL. Labeling effects were excluded by normalizing the ratio to 1∶1 in each staining group. N = 6/group. Data are combined from two independent experiments.

Next we sought to determine the impact of VAD on homing potential of allogeneic donor T cells following their activation. Therefore we adoptively transferred C57BL/6 T cells (Thy1.1^+^) into lethally irradiated allogeneic STD or VAD mice (BALB/c). Three days after transfer, VAD and STD recipient mice were sacrificed and lymphocytes were harvested from mLNs and were labeled with either CFSE or TAMRA. Mixtures of donor lymphocytes were adjusted to contain equal T cell numbers and were then intravenously transferred to untreated syngeneic (C57BL/6) recipient mice. Sixteen hours later we analyzed the distribution of T cells in pLN, mLN, spleen, liver, intraepithelial (IEL) and lamina propria lymphocytes (LPL) in the small intestine. Recovered allo-primed donor lymphocytes were identified in FACS by the congenic marker Thy1.1. The analyses revealed that intestinal homing of donor CD4^+^ (**Figure 2A**) and CD8^+^ T cells (**Figure 2B**) primed in VAD mice was reduced as compared to cells primed in STD recipients. Of note, there was no difference in homing of VAD or STD primed T cells to the non-inflamed liver in these experiments.

These competitive transfer experiments demonstrated that VAD during the priming process in the host organism was sufficient to impair homing of allo-primed T cells to the non-inflamed intestine.

### Vitamin A Deficiency in HSCT Recipients Ameliorates Acute GvHD

**Figure 3:**
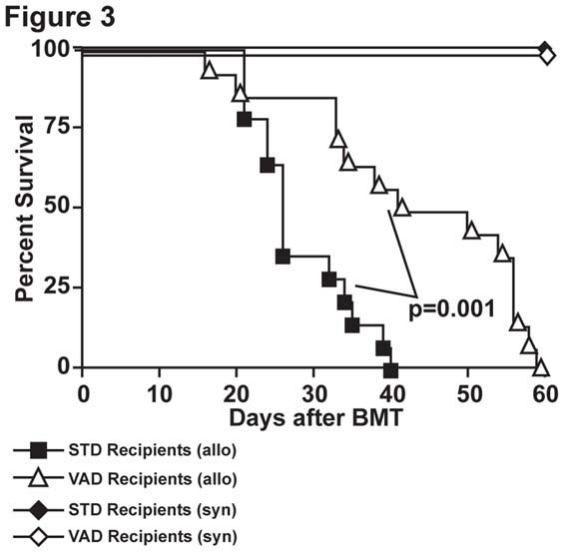
Vitamin A deficiency in HSCT recipients prolongs survival but does not protect from lethal GvHD. After lethal irradiation, STD BALB/c or VAD BALB/c recipients received 5×10^6^ C57BL/6 T cell depleted bone marrow cells supplemented with 1×10^6^ T cells (N = 14/group). STD BALB/c or VAD BALB/c recipients that received syngeneic (BALB/c) grafts were used as controls (N = 4/group). Data are combined from two independent experiments.

The intestine is the largest GvHD target organ and protection from intestinal GvHD through impaired donor T cell gut-homing has been shown to prolong survival in several models of experimental GvHD [11], [12]. Since donor T cells primed in VAD recipients were not able to home efficiently to the intestine, we investigated whether VAD recipients would be protected from development of intestinal GvHD and consequently also from lethal outcome of the disease. Therefore, lethally irradiated (800cGy) VAD or STD recipient mice (BALB/c) were transplanted with 5×10^6^ T cell depleted bone marrow (TCD-BM) from allogeneic (C57BL/6) donors along with 1×10^6^ splenic T cells from either C57BL/6 or syngeneic BALB/c donors. VAD animals were further kept under vitamin A deficient food after transplantation, whereas STD mice received standard diet throughout the duration of the experiment (60 days). Under otherwise identical conditions, VAD and STD animals transplanted with syngeneic grafts survived without any signs of acute GvHD for the time of follow up (**Figure 3**). In the groups receiving allogeneic T cells, the survival of VAD recipients was significantly prolonged when compared to STD recipients. However, all VAD mice that received allogeneic T cells, developed clinical signs of GvHD and died within 60 days after transplantation. These results suggest that altered tissue tropism of T cells prolonged survival in GvHD but since all animals died, other factors must have influenced the course of the disease.

### Donor T Cells are Reduced in Numbers in the Intestine of VAD Recipients but Accumulate in the Liver during Acute GvHD

**Figure 4:**
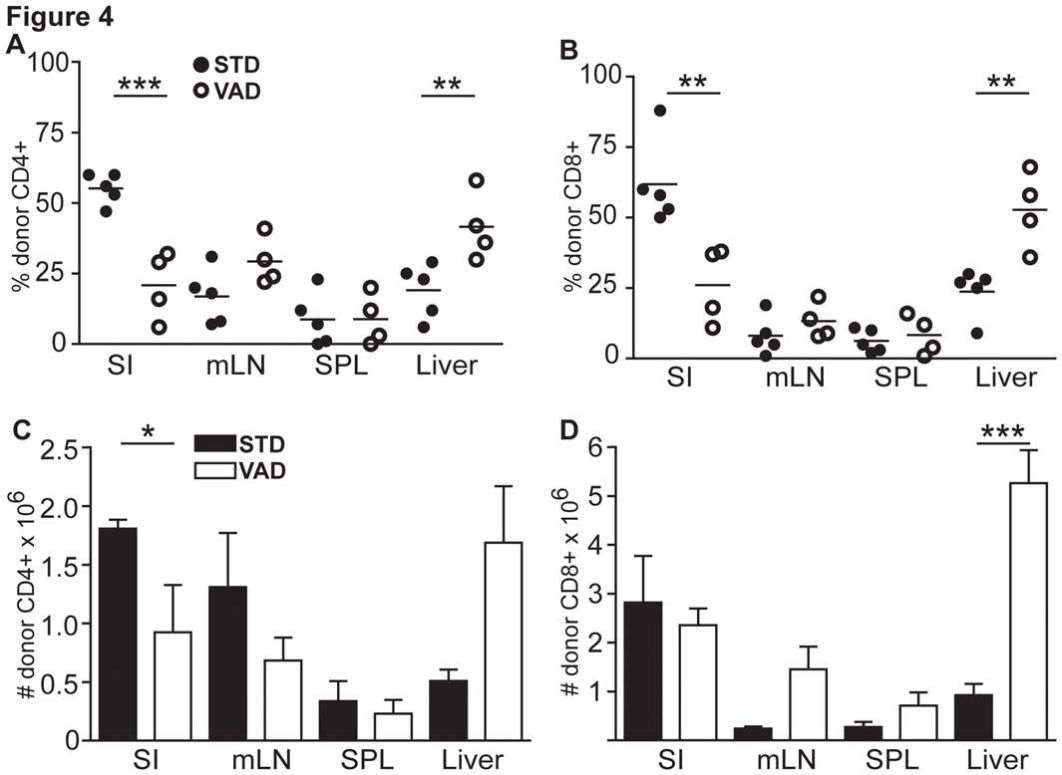
Reduced accumulation of donor T cells in the intestine and increased accumulation of donor T cells in the liver of VAD recipients during acute GvHD. Mice were analyzed for donor T cell (Thy1.1) occurrence in SPL, mLN, liver and small intestine (SI) at day 21 after transplantation. (A) Each dot represents the percentage of infiltrating Thy1.1^+^CD4^+^ T cells of all Thy1.1^+^CD4^+^ T cells. (B) Each dot represent the percentage of infiltrating Thy1.1^+^CD8^+^ T cells of all Thy1.1^+^CD8^+^ T cells. Bars indicate mean values. N = 4–5/group. **: p<0.01; ***: p<0.001. Data are combined from two independent experiments. (C) Each bar represents absolute numbers of infiltrating Thy1.1^+^CD4^+^ T cells from STD or VAD recipients. Bars indicate mean values. N = 4–5/group. **: p<0.01; ***: p<0.001. Data are combined from two independent experiments. (D) Each bar represents absolute numbers of infiltrating Thy1.1^+^CD4^+^ T cells from STD or VAD recipients. Bars indicate mean values. N = 4–5/group. **: p<0.01; ***: p<0.001. Data are combined from two independent experiments.

To test whether homing of donor T cells is altered under VAD conditions during GvHD, we assessed the infiltration of donor CD4^+^ and CD8^+^ T cell into GvHD-target organs. To that end, liver, small bowel, mLN, and spleen of the recipients were harvested at different time points after transplantation and the percentage of infiltrating CD4^+^ and CD8α^+^ donor T cells (Thy1.1^+^) was determined by FACS analysis. Interestingly, when analyzing homing of donor T cells to GvHD target tissues under highly inflammatory conditions early after transplantation we failed to detect differences in VAD as compared to STD recipients. However, in line with our initial hypothesis we observed significantly reduced frequencies of donor CD4^+^ and CD8^+^ T cells in the intestines of VAD as compared to STD recipients three weeks after transplantation (**Figure 4A, B**). At that time we also observed significantly higher frequencies of CD8^+^ and CD4^+^ donor T cells in the livers of VAD recipients. There was no difference in CD4^+^ or CD8^+^ T cell frequencies in mLN or spleen detected in both groups (**Figure 4A, B**). When analyzing absolute numbers of donor CD4^+^ and CD8^+^ T cell in GvHD target organs we observed less accumulation of CD4^+^, but not of CD8^+^ T cells in small intestines of VAD-recipients. Although there was a clear trend, CD4^+^ T cell numbers were not significantly increased in livers of VAD recipients, whereas we observed significantly higher CD8^+^ T cell counts in this organ (**Figure 4C, D**). These results indicate that lack of dietary vitamin A alters migration routes of donor T-cells in experimental GvHD, but also suggest that mechanisms other than altered homing to the intestine contribute to a serious damage of other target organs of GvHD such as the liver.

### VAD Alters Donor T Cell Polarization into Th1 and Treg Cells

**Figure 5:**
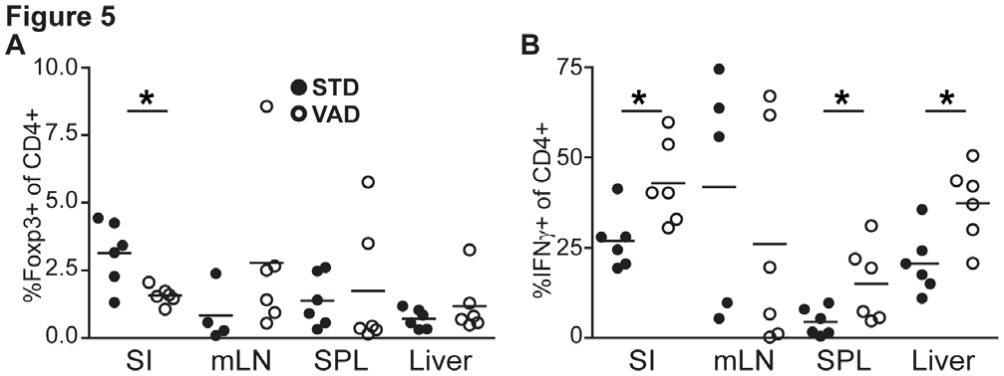
Vitamin A deficiency of recipient mice leads to increased Th1 cells and decreased FoxP3^+^ Treg cells during GvHD. STD or VAD recipient mice were analyzed for CD4^+^ T cell polarization status in SPL, mLN, liver and small intestine (SI) at day 21 after transplantation. (**A**) Each bar represents the percentage of FoxP3^+^ CD4^+^ T cells of CD4^+^ T cells isolated from the indicated organ. (**B**) Each bar represents the percentage of IFN-γ^+^ CD4^+^ T cells of CD4^+^ T cells isolated from the indicated organ.

It has been reported that VAD can alter CD4^+^ T cell polarization [14], [15]. Therefore we speculated that this also influences the observed phenotype in GvHD in VAD recipients. To test this, we employed intracellular cytokine (IFN-γ, IL-4 and IL-17) and FoxP3-stainings to analyze CD4^+^ T cell lineages after allogeneic transplantation in target organs of GvHD by flow cytometry. We found significantly reduced frequencies of CD4^+^FoxP3^+^ Treg cells in small intestines of VAD as compared to STD recipient mice (**Figure 5A**). In line with findings in other animal models [14], [15], we observed significantly higher frequencies of IFN-γ-producing CD4^+^ T cells in small intestines, spleen and livers of VAD as compared to STD recipients (**Figure 5B**). We did not observe marked differences in Th2 and Th17 cells when comparing both groups. Taken together these results suggest that VAD conditions lead to reduced numbers of Treg cells and more Th1 cells in target organs of GvHD. Thus, our observations suggested an enhanced pro-inflammatory scenario due to the absence of vitamin A in this model.

### Vitamin A Deficiency Leads to Increased Hepatic Inflammation

**Figure 6:**
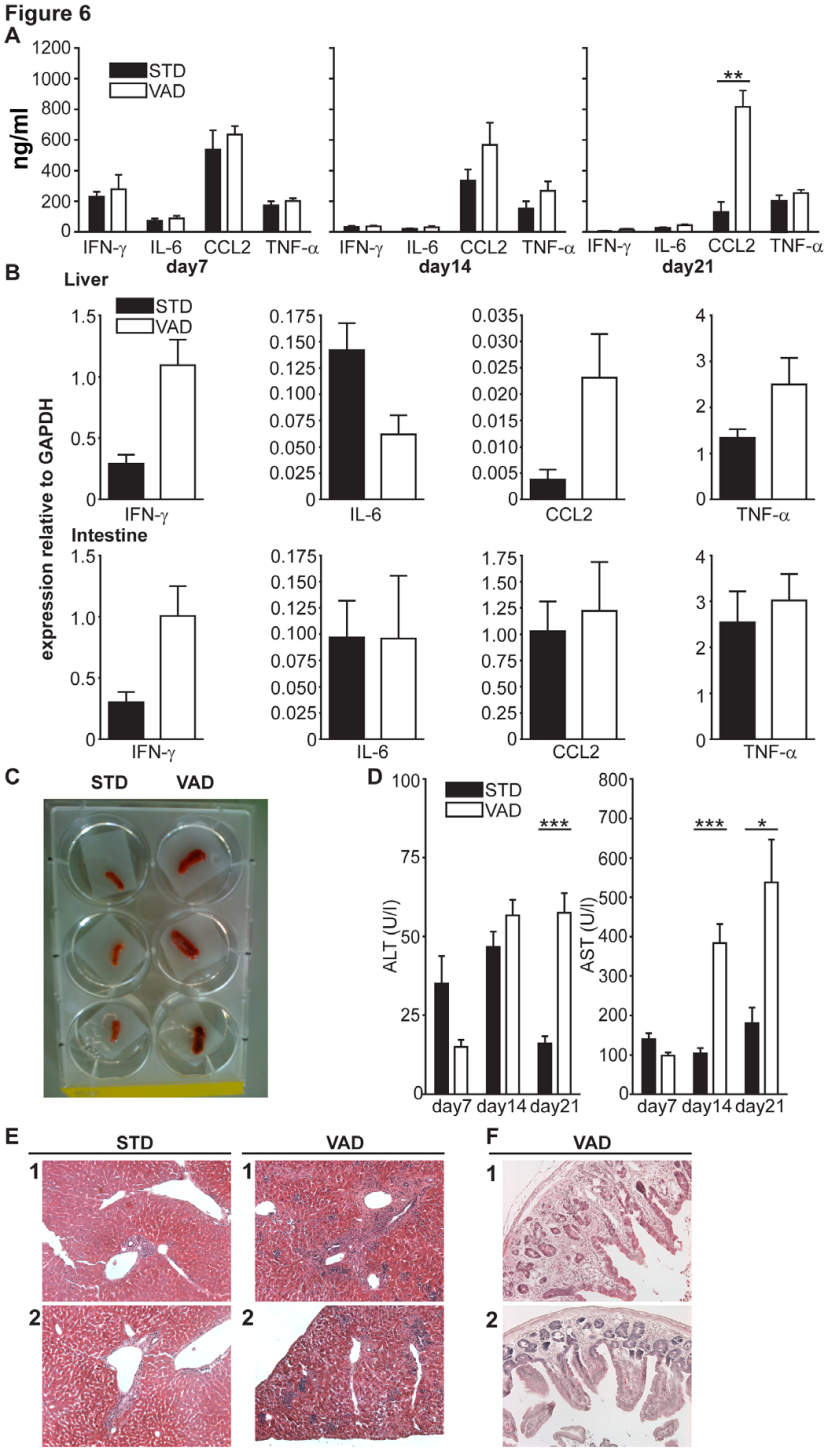
Vitamin A deficiency led to increased hepatic inflammation. (**A**) Serum cytokine levels were analyzed using the Cytometric Bead Array, Mouse Inflammation Kit (BD Biosciences). N = 5–6/group. **: p<0.01; ***: p<0.001. Data are combined from two independent experiments. (**B**) Expression analyses of cytokine levels of GvHD target organs using RT-PCR (N = 6/group) Data are combined from two independent experiments (**C**) Macroscopic comparison of spleen sizes of VAD and STD recipient mice. N = 3/group. One of two representative experiments is shown. (**D**) Liver enzymes in the course of GvHD in VAD and STD recipients. N = 5–6/group. **: p<0.01; ***: p<0.001. Data are combined from two independent experiments. (**E, F**) Representative sections from formalin fixed, paraffin embedded livers and small intestines harvested at 21 days post transplantation. The 4–6 µm slides were stained with hematoxylin and eosin.

To estimate systemic inflammation in the course of the disease we analyzed inflammatory cytokines in the serum of VAD and STD recipients every week after transplantation. Serum levels of the pro-inflammatory cytokines interferon-γ (IFN-γ, IL-6, chemokine C-C motif ligand 2 (CCL2) and tumor necrosis factor α (TNF-α) were used as global markers for inflammation. Whereas serum levels of the pro-inflammatory cytokines did not markedly differ between STD and VAD animals during the first two weeks after allogeneic HSCT, we observed a significant increase of CCL2 serum concentrations in VAD recipients as compared to STD recipients in the third week (**Figure 6A**). To localize the source of elevated cytokines and chemokines measured in the serum we analyzed expression of these proteins in liver and intestine in both groups after transplantation. In the liver of VAD recipients we observed higher expression of IFN-γ, CCL2 and TNF levels, whereas we found up-regulation of IL-6 in STD-recipients. We also found stronger IFN-γ-expression in the intestines of VAD-recipients as compared to STD-mice (**Figure 6B**). Three weeks after transplantation, when we also observed high levels of CCL2, we found enlarged spleens in VAD recipients as depicted in **Figure 6C**.

Due to the observed accumulation of donor T cells in the liver of VAD mice during GvHD, we measured the liver enzymes alanine aminotransferase (ALT) and aspartate aminotransferase (AST) in the serum as an indicator for liver injury. VAD recipients showed increased serum concentrations of AST at day 14 and an increased concentration of both transaminases, AST and ALT, at day 21 after transplantation as compared to STD recipients (**Figure 6D**). Increased serum concentrations of liver enzymes such as AST and ALT are indicative of liver cell damage during GvHD [16]. In line with elevated serum levels of liver enzymes, we observed extensive signs of tissue damage and cell infiltration in livers of VAD recipients as compared to STD recipients (**Figure 6E**), at the same time tissue damage was low in small intestines of VAD recipients (**Figure 6F**). Taken together these data suggest that Vitamin A deficiency led to a global inflammation in recipient mice with severe liver damage. We assume that liver damage together with other secondary effects of VAD finally led to fatal GvHD.

## Discussion

The intestine is the major target organ of acute GvHD, which frequently accounts for life threatening complications. Assault of the intestine by alloreactive T cells requires their access to the tissue. Thus, diversion of T cell migration away from the intestine is likely to ameliorate the clinical outcome in acute GvHD.

Iwata and colleagues identified the Vitamin A metabolite RA as the main inducer for gut-homing receptors on T cells [8]. Further studies showed that not only DCs but also stroma cells are providers for RA in GALT [10]. Interestingly, mice raised under Vitamin A deficient conditions contained fewer α4β7^+^ T cells than mice fed with standard diet and these cells failed to efficiently home to the intestine under steady state conditions [8]. Therefore, we explored deprivation of Vitamin A and subsequent inhibition of gut-homing in acute GvHD.

We showed that acquisition of integrin-α4β7 and CCR9 on allogeneic T cells during GvHD priming phase was dependent on Vitamin A in the recipients’ diet. In the course of acute GvHD, we saw diminished accumulation of CD4^+^ and CD8^+^ donor T cells in the intestine of VAD recipients when compared to STD recipients. Importantly, we also found that lack of dietary Vitamin A prolonged survival in experimental GvHD.

Of interest, the major physiologic metabolite of vitamin A, all-*trans*-RA (ATRA), is given to patients for treatment of acute myeloid leukemia (AML) [17] and has been shown to exert significant effects on the expression of integrin-α4β7 *in vitro* [8]. Of note, AML is currently the most frequent indication for allogeneic HSCT [18]. Once ATRA-treated patients are referred to allogeneic HSCT, they have high levels of vitamin A. Therefore, it is conceivable that high levels of vitamin A could lead to an increased gut-homing phenotype of T cells and thus ATRA-treatment might have an influence on the development of intestinal GvHD after HSCT. Mechanistically, vitamin A may control expression of retinal dehydrogenase enzymes in mucosal dendritic cells and gut-draining lymph node stromal cells as recently shown by Hammerschmidt [19] and Molenaar [20].

However, despite reduced intestinal T cell homing, recipient mice fed with vitamin A deficient diet were not protected from lethal GvHD. In this context, it is also important to note that the induction and expansion of regulatory T cells under homeostatic conditions and during intestinal inflammation is dependent on vitamin A [21]–[23]. Treg cells are of major importance for reduction of GvHD severity [24]. Therefore we analyzed the frequencies and absolute numbers of Treg cells in the course of GvHD. As expected, we observed lower frequencies of FoxP3^+^ Treg cells after transplantation in the intestines of VAD as compared to STD recipients. These results suggested that vitamin A is necessary for migration, induction and/or expansion of Treg cells in the gut also during acute GvHD. This result is in line with recent findings showing that homing, induction and stability of FoxP3^+^CD4^+^ T cells depends on vitamin A [23], [25]. However, our survival data indicated that a potential beneficial effect of Treg numbers can be overcome by adverse mechanisms such as altered tissue tropism of effector T cells. Furthermore, Treg-mediated effects influenced by RA are probably more important in chronic diseases as previously reported by others [25]. This is in line with previous work from our group showing that induced Treg cells do not protect from acute GvHD in this model [26]. However, we observed higher frequencies of Th1 T cells when analyzing the lineage differentiation of donor CD4^+^ T lymphocytes in target organs of GvHD in VAD as compared to STD recipients (**Figure 5**). This was also consistent with increased IFN-γ expression as measured by RT-PCR in liver and intestine tissue samples after GvHD (**Figure 6B**). It has been shown in different animal models that vitamin A deficiency promotes Th1 polarization [14], [15]. We confirmed these observations in the present study. Since the B6BALB/c acute GvHD model is a Th1 type inflammatory disease [27] the higher frequency of IFN-γ producing CD4^+^ T cells might have also contributed to the final outcome of the disease.

Furthermore, liver enzymes, cytokine expression pattern and histology revealed severe hepatic GvHD in VAD animals. In line with this we observed strong accumulation of donor T cells in the livers of VAD animals after transplantation. Interestingly, we also found higher expression levels of the chemokine CCL2 and increased CD8^+^ T cell numbers in the livers of VAD recipients. CCL2 is known to be expressed in the liver during GvHD [28] and attracts CCR2^+^CD8^+^ T cells to the liver as shown previously by others [29]. Therefore, we speculate that such inflammatory chemokines might also contribute to the disease outcome of GvHD under VAD conditions. Considering that the liver is the largest vitamin A storage of the organism [30], we can not exclude that factors other than increased homing of alloreactive T cells to the liver might also contribute to the observed liver damage in VAD recipients. In addition to its role in the immune system, vitamin A deficiency has been shown to affect several functions of vertebrates including reproduction, epithelial cell and bone marrow differentiation and vision.

In conclusion, our study shows that induction of gut-homing potential of allogeneic donor T cells relies on the presence of vitamin A in the host organism. We also show that lack of this nutrient has multiple secondary effects that trigger acute inflammation with severe hepatic damage in this model of acute GvHD.

## Materials and Methods

### Ethics Statement

All animal experiments were done in accordance with institutional and governmental directives and were approved by the institutional review board and the “Niedersächsisches Landesamt für Verbraucherschutz und Lebensmittelsicherheit” (protocol #07/1383).

### Mice

C57BL/6 (H-2K^b^) Thy1.2^+^, C57BL/6 Thy1.1^+^ and BALB/c (H-2K^d^) mice were bred at the central animal facility of Hannover Medical School under specific pathogen-free conditions or purchased from Charles River Laboratories (Sulzfeld, Germany). Vitamin A deficient BALB/c (H-2K^d^) mice were prepared by feeding them a vitamin A depleted diet from gestational day 14 [8]. Control animals received standard (STD) diet. Litters were weaned at four weeks of age and maintained on the same diet at least upon completion of experiments.

### Antibodies and Flow Cytometric Analysis (FACS)

FACS data were acquired on LSRII (BD-Biosciences, San Jose, CA) and analyzed using FlowJo software (Treestar, Ashland, OR). The rat anti-mouse CCR9 (clone 7E7-1-1) was produced with rat hybridoma cell lines [31]. The anti-mouse integrin-β7-PE (clone LPAM-1), anti-Thy1.1-PE, CD4-PerCP, CD8-APC-Cy7 and CD62L-FITC were purchased from BD Biosciences (San Jose, CA). Biotinylated antibodies were recognized by strepatvidin coupled to Alexa Fluor 405 or PE (both Invitrogen, Carlsbad, CA). Rat and mouse sera were purchased from AbD Serotec (Oxford, U.K.) and Invitrogen (Carlsbad, CA), respectively. For measurements of intracellular cytokines, T cells were stimulated with 50 ng/mL Phorbol-12-myristate-13-acetate (PMA, Calbiochem, Darmstadt, Germany), 2 µg/mL ionomycin (Invitrogen, Camarillo, CA) for 1 h, followed by further 3 h incubation in the presence of 1 µg/mL brefeldin A (Sigma-Aldrich, St. Louis, MI). Cells were fixed using a Fix/Perm buffer set (eBioscience, San Diego, CA) as described in the suppliers’ manual. For intracellular stainings, we used anti-FoxP3-APC (clone FJK-16s, eBiosciences, San Diego, CA), anti-IL-17A-PE (clone TC11-18H10, BD-Biosciences, San Jose, CA), IL-4-APC (clone 11B11, BD-Biosciences, San Jose, CA) and anti-IFN-γ-PE (XMG1.2, BioLegend or eBiosciences, both San Diego, CA).

### Analysis of T Cell Proliferation in vivo

Single-cell suspensions were prepared from red blood cell (RBC)-lysed splenocytes by treatment with hypotonic NH4Cl, and subsequently stained with 5 µM carboxyfluorescin diacetate succinimidyl ester (CFSE; Molecular Probes, Eugen, OR) for 15 minutes at 37°C. 2×10^7^ cells were transplanted into lethally irradiated BALB/c mice. Recipient mice were sacrificed after 72 hours and SLOs were harvested and analyzed by FACS.

### Competitive Homing Experiments

Single-cell suspensions were prepared from mLNs of BALB/c mice fed with STD or VAD food at day 3 after HSCT. After RBC-lysis, cells were labeled with 10 µM 5-(and-6-)-TAMRA SE or 5 µM CFSE (Invitrogen, Carlsbad, CA) for 15 min at 37°C. C57BL/6 recipient mice (6–8 weeks old) received 5×10^6^ labeled T cells in a ratio of 1∶1, VAD and STD by intravenous injection into the lateral tail vein. Sixteen hours after transfer C57BL/6 recipient mice were sacrificed and lymphocytes were harvested for assessment of organ infiltration.

### Bone Marrow Transplantation

Lymphocytes were prepared from RBC-lysed splenocytes. The samples were enriched for T cells using magnetic microbeads (MACS, Pan-T cell isolation kit, Miltenyi Biotec, Auburn, CA). The bone marrow cells were harvested from femur and tibia and after RBC-lysis, T cells were stained with anti-TCRβ biotin (clone H57-597, BD Biosciences, San Jose, CA) and then depleted by MACS with strepatvidin-magnetic beads (Miltenyi Biotec, Auburn, CA). The C57BL/6 (H-2^b^) into BALB/c (H-2^d^) acute GvHD models were performed as described elsewhere [26], [32]. In brief, BALB/c recipients were lethally irradiated with 800cGy or from a ^137^Cs γ-Source. Donor cells were injected via tail vein at the same day. All recipient mice received 5×10^6^ T cell MACS-depleted (C57BL/6 or BALB/c) bone marrow cells and MACS-sorted 1×10^6^ C57BL/6 T cells. After HSCT, mice were kept on antibiotic drinking water (Cotrimoxazol, Ratiopharm, Ulm, Germany) for the first three weeks. Survival, weight loss and clinical signs of GvHD of recipient mice were monitored daily. Mice receiving vitamin A depleted diet or control diet had equivalent body weights at time of transplantation.

### Assessment of GvHD Target Organ Infiltration and Histology

Recipient mice were sacrificed at different time points after HSCT and organs (spleen, liver, pLN, mLN, small intestine) were harvested for assessment of organ infiltration with donor T cells. Single-cell suspensions of lymph nodes and spleen were obtained by mincing the tissue through a nylon mesh. For isolation of LPL and IEL, gut content and PPs were removed before intestines were opened longitudinally. Intestines were washed twice in cold PBS and once in cold PBS/5%FCS/ 5mM EDTA, and incubated twice in RPMI1640 medium supplemented with 5%FCS and 5 mM EDTA at 37°C. Supernatants were pooled, filtered through a nylon mesh, pelleted and resuspended (IEL fraction) [33]. The remaining tissue was incubated twice at 37°C for 45 minutes in RPMI1640 with 10% FCS, 0.24 mg/ml collagenase A (Roche, Grenzach-Wyhlen, Germany) and 40 U/ml DNAse I (Roche, Grenzach-Wyhlen, Germany). Again, supernatants were pooled, filtered through a mesh, pelleted and resuspended (LPL fraction) [21]. Liver lymphocytes were obtained after perfusion of the livers. Then organs were cut into small pieces, then incubated in RPMI 1640 medium supplemented with 10% FCS, 25 mM HEPES, 0.25 mg/ml collagenase IV, and 12.5 mg/ml DNase I (Sigma-Aldrich) for 45 min at 37°C. After incubation, enzymatic activity was terminated by addition of EDTA to a final concentration of 20 mM. The medium was then filtered through a nylon mesh [34]. Lymphocytes were recovered from intestinal (IEL and LPL) and liver samples by density gradient centrifugation on Percoll (Amersham Biosciences). Pooled lymphocytes from the IEL and LPL fraction represent the total lymphocytes of SI in our experiments. Samples from spleen, and liver were subjected to RBC lysis.

### Cytokine Bead Array and Liver Function Tests

Peripheral blood was obtained at different time points from treated or untreated mice. Serum was separated, and concentrations of AST and ALT were measured by an automated method using an Olympus AU 400 analyzer (Beckman Coulter, Inc., Krefeld, Germany). Serum levels of the pro-inflammatory cytokines IL-6, CCL2, TNFα and IFN-γ were quantified by bead-based flow cytometry assay (“mouse inflammatory cytokine cytometric bead array kit”, Becton Dickinson, Franklin Lakes, New Jersey, USA) according to the manufacturer’s protocol.

### Determination of RNA Expression Levels with RT-PCR of Liver and Intestine

Cellular RNA was extracted from snap frozen recipient 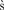 liver or intestinal samples using TRIZOL (Invitrogen, Carlsbad, CA) according to the manufacturer’s protocol and first strand cDNA was synthesized from 1 µg DNase treated total RNA. Real-time PCR reactions were carried out on a StepOne Plus real-time PCR system (Applied Biosystems, Foster City, CA, USA) by using the *Mcpt1, IL6, IFNg, Tnf* TaqMan gene expression assays (Assay IDs: *Mcpt1*: Mm00656886_g1; *IL6*: Mm00446190_m1; *Ifng*: Mm01168134_m1; *Tnf*: Mm00443260_g1). GAPDH was used as control (TaqMan Rodent GAPDH Control Reagents, Applied Biosystems, Foster City, CA, USA).

### Statistical Analysis

Statistical analysis was performed with Prism 4 (Graph-Pad Software, Inc.). Significant values were determined using the unpaired two-tailed t test. Statistical differences for the mean values are as follows: *, P≤0.05; **, P≤0.01; and ***, P≤0.001. To analyze survival, Kaplan-Meier estimation and log-rank test was used.

## References

[1] Socie G, Blazar BR (2009). Acute graft-versus-host disease: from the bench to the bedside.. Blood.

[2] Beilhack A, Schulz S, Baker J, Beilhack GF, Wieland CB (2005). In vivo analyses of early events in acute graft-versus-host disease reveal sequential infiltration of T-cell subsets.. Blood.

[3] SACKSTEIN R (2006). A Revision of Billingham’s Tenets: The Central Role of Lymphocyte Migration in Acute Graft-versus-Host Disease.. Biology of Blood and Marrow Transplantation.

[4] Agace WW (2008). T-cell recruitment to the intestinal mucosa.. http://dx.doi.org/10.1016/j.it.2008.08.003.

[5] Wurbel MA, Philippe JM, Nguyen C, Victorero G, Freeman T (2000). The chemokine TECK is expressed by thymic and intestinal epithelial cells and attracts double- and single-positive thymocytes expressing the TECK receptor CCR9. Eur J Immunol 30: 262–271.. doi:10.1002/1521-4141(200001)30:1<262::AID-IMMU262>3.0.CO;.

[6] Kunkel EJ, Campbell JJ, Haraldsen G, Pan J, Boisvert J (2000). Lymphocyte CC chemokine receptor 9 and epithelial thymus-expressed chemokine (TECK) expression distinguish the small intestinal immune compartment: Epithelial expression of tissue-specific chemokines as an organizing principle in regional immunity.. J Exp Med.

[7] Campbell DJ, Butcher EC (2002). Rapid acquisition of tissue-specific homing phenotypes by CD4(+) T cells activated in cutaneous or mucosal lymphoid tissues.. J Exp Med.

[8] Iwata M, Hirakiyama A, Eshima Y, Kagechika H, Kato C (2004). Retinoic acid imprints gut-homing specificity on T cells.. Immunity.

[9] Iliev ID, Mileti E, Matteoli G, Chieppa M, Rescigno M (2009). Intestinal epithelial cells promote colitis-protective regulatory T-cell differentiation through dendritic cell conditioning.. Mucosal Immunol.

[10] Hammerschmidt SI, Ahrendt M, Bode U, Wahl B, Kremmer E (2008). Stromal mesenteric lymph node cells are essential for the generation of gut-homing T cells in vivo.. J Exp Med.

[11] Petrovic A, Alpdogan O, Willis LM, Eng JM, Greenberg AS (2004). LPAM (alpha 4 beta 7 integrin) is an important homing integrin on alloreactive T cells in the development of intestinal graft-versus-host disease.. Blood.

[12] Waldman E, Lu SX, Hubbard VM, Kochman AA, Eng JM (2006). Absence of beta7 integrin results in less graft-versus-host disease because of decreased homing of alloreactive T cells to intestine.. Blood.

[13] Beilhack A, Schulz S, Baker J, Beilhack GF, Nishimura R (2008). Prevention of acute graft-versus-host disease by blocking T-cell entry to secondary lymphoid organs.. Blood.

[14] Schuster GU, Kenyon NJ, Stephensen CB (2008). Vitamin A deficiency decreases and high dietary vitamin A increases disease severity in the mouse model of asthma.. J Immunol.

[15] Cantorna MT, Nashold FE, Hayes CE (1994). In vitamin A deficiency multiple mechanisms establish a regulatory T helper cell imbalance with excess Th1 and insufficient Th2 function.. J Immunol.

[16] Yasmineh WG, Filipovich AH, Killeen AA (1989). Serum 5′nucleotidase and alkaline phosphatase–highly predictive liver function tests for the diagnosis of graft-versus-host disease in bone marrow transplant recipients.. Transplantation.

[17] Tallman MS (2005). New strategies for the treatment of acute myeloid leukemia including antibodies and other novel agents.. http://dx.doi.org/10.1182/asheducation-2005.1.143.

[18] Gooley TA, Chien JW, Pergam SA, Hingorani S, Sorror ML (2010). Reduced mortality after allogeneic hematopoietic-cell transplantation.. N Engl J Med.

[19] Hammerschmidt SI, Friedrichsen M, Boelter J, Lyszkiewicz M, Kremmer E, et al (2011). Retinoic acid induces homing of protective T and B cells to the gut after subcutaneous immunization in mice.. J Clin Invest.

[20] Molenaar R, Knippenberg M, Goverse G, Olivier BJ, de Vos AF (2011). Expression of Retinaldehyde Dehydrogenase Enzymes in Mucosal Dendritic Cells and Gut-Draining Lymph Node Stromal Cells Is Controlled by Dietary Vitamin A. The Journal of Immunology. http://dx.doi.org/10.4049/jimmunol.1001672.

[21] Hadis U, Wahl B, Schulz O, Hardtke-Wolenski M, Schippers A (2011). Intestinal Tolerance Requires Gut Homing and Expansion of FoxP3+ Regulatory T Cells in the Lamina Propria.. Immunity.

[22] Menning A, Loddenkemper C, Westendorf AM, Szilagyi B, Buer J (2010). Retinoic acid-induced gut tropism improves the protective capacity of Treg in acute but not in chronic gut inflammation.. http://dx.doi.org/10.1002/eji.200939938.

[23] Kang SG, Wang C, Matsumoto S, Kim CH (2009). High and low vitamin A therapies induce distinct FoxP3+ T-cell subsets and effectively control intestinal inflammation.. Gastroenterology.

[24] Hoffmann P, Ermann J, Edinger M, Fathman CG, Strober S (2002). Donor-type CD4(+)CD25(+) regulatory T cells suppress lethal acute graft-versus-host disease after allogeneic bone marrow transplantation.. J Exp Med.

[25] Zhou X, Kong N, Wang J, Fan H, Zou H (2010). Cutting Edge: All-Trans Retinoic Acid Sustains the Stability and Function of Natural Regulatory T Cells in an Inflammatory Milieu.. The Journal of Immunology.

[26] Koenecke C, Czeloth N, Bubke A, Schmitz S, Kissenpfennig A (2009). Alloantigen-specific de novo-induced Foxp3+ Treg revert in vivo and do not protect from experimental GVHD.. Eur J Immunol.

[27] Reddy PR, Negrin RS, Hill GR (2008). Mouse models of bone marrow transplantation.. Biol Blood Marrow Transplant.

[28] Ichiba T, Teshima T, Kuick R, Misek DE, Liu C (2003). Early changes in gene expression profiles of hepatic GVHD uncovered by oligonucleotide microarrays.. Blood.

[29] Terwey TH, Kim TD, Kochman AA, Hubbard VM, Lu S (2005). CCR2 is required for CD8-induced graft-versus-host disease.. Blood.

[30] Harrison EH (2005). MECHANISMS OF DIGESTION AND ABSORPTION OF DIETARY VITAMIN A*.. Annu Rev Nutr.

[31] Pabst O, Ohl L, Wendland M, Wurbel M-A, Kremmer E (2004). Chemokine receptor CCR9 contributes to the localization of plasma cells to the small intestine.. J Exp Med.

[32] Seth S, Ravens I, Lee C-W, Glage S, Bleich A (2011). Absence of CD155 aggravates acute graft-versus-host disease.. Proc Natl Acad Sci USA.

[33] Koenecke C, Chennupati V, Schmitz S, Malissen B, Foerster R (2009). In vivo application of mAb directed against the gammadelta TCR does not deplete but generates “invisible” gammadelta T cells.. Eur J Immunol.

[34] Seth S, Oberdorfer L, Hyde R, Hoff K, Thies V (2011). CCR7 Essentially Contributes to the Homing of Plasmacytoid Dendritic Cells to Lymph Nodes under Steady-State As Well As Inflammatory Conditions.. The Journal of Immunology.

